# Deep Convolutional Neural Networks for Chest Diseases Detection

**DOI:** 10.1155/2018/4168538

**Published:** 2018-08-01

**Authors:** Rahib H. Abiyev, Mohammad Khaleel Sallam Ma'aitah

**Affiliations:** Department, of Computer Engineering, Near East University, North Cyprus, Mersin-10, Turkey

## Abstract

Chest diseases are very serious health problems in the life of people. These diseases include chronic obstructive pulmonary disease, pneumonia, asthma, tuberculosis, and lung diseases. The timely diagnosis of chest diseases is very important. Many methods have been developed for this purpose. In this paper, we demonstrate the feasibility of classifying the chest pathologies in chest X-rays using conventional and deep learning approaches. In the paper, convolutional neural networks (CNNs) are presented for the diagnosis of chest diseases. The architecture of CNN and its design principle are presented. For comparative purpose, backpropagation neural networks (BPNNs) with supervised learning, competitive neural networks (CpNNs) with unsupervised learning are also constructed for diagnosis chest diseases. All the considered networks CNN, BPNN, and CpNN are trained and tested on the same chest X-ray database, and the performance of each network is discussed. Comparative results in terms of accuracy, error rate, and training time between the networks are presented.

## 1. Introduction

Medical X-rays are images which are generally used to diagnose some sensitive human body parts such as bones, chest, teeth, skull, and so on. Medical experts have used this technique for several decades to explore and visualize fractures or abnormalities in body organs [1]. This is due to the fact that X-rays are very effective diagnostic tools in revealing the pathological alterations, in addition to its noninvasive characteristics and economic considerations [2]. Chest diseases can be shown in CXR images in the form of cavitations, consolidations, infiltrates, blunted costophrenic angles, and small broadly distributed nodules [3]. By analyzing the chest X-ray image, the radiologists can diagnose many conditions and diseases such as pleurisy, effusion, pneumonia, bronchitis, infiltration, nodule, atelectasis, pericarditis, cardiomegaly, pneumothorax, fractures, and many others [4].

Classifying the chest X-ray abnormalities is considered as a tedious task for radiologists; hence, many algorithms were proposed by researchers to accurately perform this task [5–7]. Over the past decades, computer-aided diagnosis (CAD) systems have been developed to extract useful information from X-rays to help doctors in having a quantitative insight about an X-ray. However, these CAD systems could not have achieved a significance level to make decisions on the type of conditions of diseases in an X-ray [2–4]. Thus, the role of them was left as visualization functionality that helps doctors in making decisions.

A number of research works have been carried out on the diagnosis of chest diseases using artificial intelligence methodologies. In [1], multilayer, probabilistic, learning vector quantization, and generalized regression neural networks have been used for diagnosis chest diseases. The diagnosis of chronic obstructive pulmonary and pneumonia diseases was implemented using neural networks and artificial immune system [8]. In [9], the detection of lung diseases such as TB, pneumonia, and lung cancer using chest radiographs is considered. The histogram equalization in image segmentation was applied for image preprocessing, and feedforward neural network is used for classification purpose. The above research works have been efficiently used in classifying medical diseases; however, their performance was not as efficient as the deep networks in terms of accuracy, computation time, and minimum square error achieved. Deep learning-based systems have been applied to increase the accuracy of image classification [10, 11]. These deep networks showed superhuman accuracies in performing such tasks. This success motivated the researchers to apply these networks to medical images for diseases classification tasks, and the results showed that deep networks can efficiently extract useful features that distinguish different classes of images [12–15]. Most commonly used deep learning architecture is the convolutional neural network (CNN). CNN has been applied to various medical images classification due to its power of extracting different level features from images [11–15].

**Figure 1:**
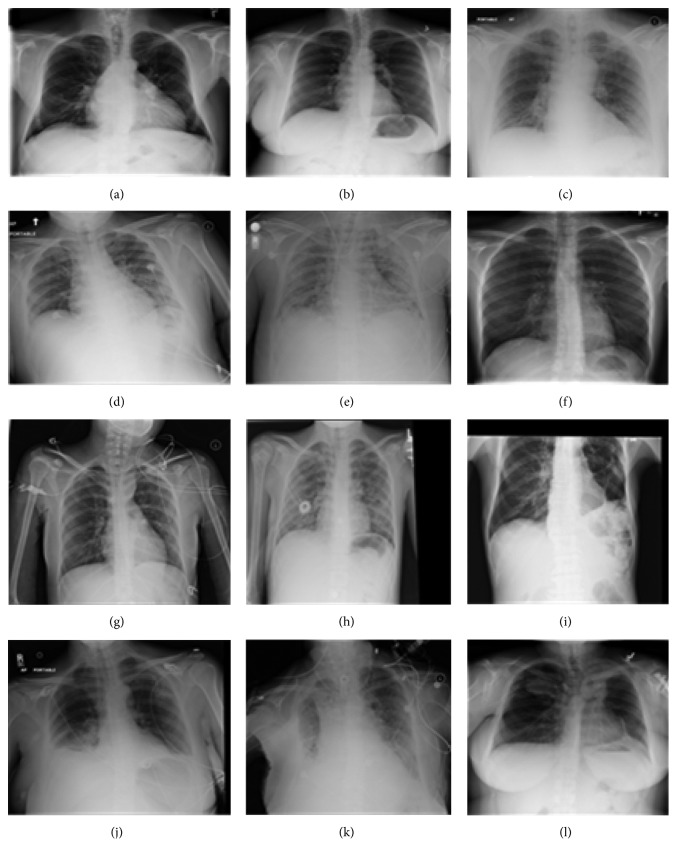
Chest X-ray diseases. (a) Atelectasis. (b) Cardiomegaly. (c) Consolidation. (d) Edema. (e) Effusion. (f) Emphysema. (g) Fibrosis. (h) Infiltration. (i) Mass. (j) Nodule. (k) Pneumonia. (l) Pneumothorax.

Having gone through the related research studies, in this paper, a deep convolutional neural network (CNN) is employed to improve the performance of the diagnosis of the chest diseases in terms of accuracy and minimum square error achieved. For this purpose, traditional and deep learning-based networks are employed to classify most common thoracic diseases and to present comparative results. Backpropagation neural network (BPNN), competitive neural network (CpNN), and convolutional neural network (CNN) are examined to classify 12 common diseases that may be found in the chest X-ray, that is, atelectasis, cardiomegaly, effusion, infiltration, mass, nodule, pneumonia, pneumothorax, consolidation, edema, emphysema, and fibrosis (Figure 1). In this paper, we aim at training both traditional and deep network using the same chest X-ray dataset and evaluating their performances. The data used in the paper are obtained from the National Institutes of Health—Clinical Center [16]. The dataset contains 112,120 frontal-view X-ray images of 30,805 unique patients.

This paper is structured as follows: Section 2 presents the methodologies used for diagnosis chest diseases. A brief explanation of the BPNN, CpNN, and CNN is given. A description of the convolutional neural network used for diagnosis chest diseases and its operating principles are presented.  Section 3 discusses the results of simulations of the networks used, in addition to the database description. A comparison of the performances of the networks used in simulations is given in Section 4, and Section 5 is the conclusion part of the paper.

## 2. Machine Learning for Diagnosis of Chest Diseases

### 2.1. Backpropagation Neural Network (BPNN)

**Figure 2:**
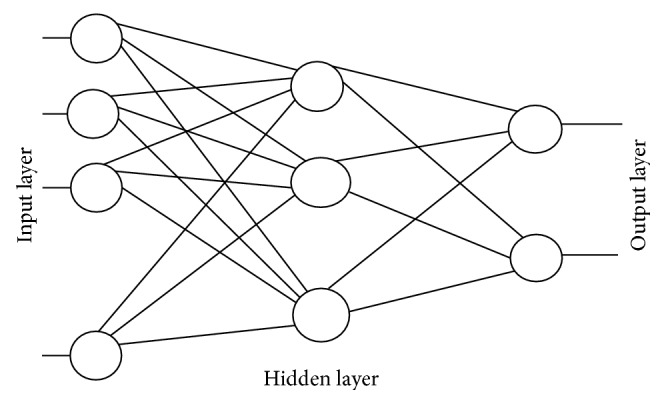
Backpropagation neural network.

Backpropagation neural network (BPNN) is a multilayer feedforward neural network that uses a supervised learning algorithm known as error back-propagation algorithm. Errors accumulated at the output layer are propagated back into the network for the adjustment of weights [16–19].  Figure 2 depicts a conventional BPNN which consists of three layers: input, hidden, and output. As seen in Figure 2, there is no backward pass of computation except the operations used in training. All the functioning operations proceed in the forward direction during simulation.

The pseudocode algorithm for BPNN is given below [20].

- Network initialization: randomly choose the initial weights
- Select first training pair
- Forward computation that includes the following steps:
  - Apply the inputs to the network
  - Calculate the output for every neuron from the input layer, through the hidden layer(s), to the output layer
  - Calculate the error at the outputs
- Backward computation
  - Use the output error to compute error signals for preoutput layers
  - Use the error signals to compute weight adjustments
  - Apply the weight adjustments
- Repeat Forward and Backward computations for other training pairs.
- Periodically evaluate the network performance. Repeat Forward and Backward computations until the network converges on the target output.

To calculate outputs for each neuron based on the input pattern, the equations below can be used. The output of the *j*-th neuron for the pattern *p* is *O*_*pj*_:

(1) $$\begin{array}{c} O_{pj}\left({\text{net}}_{j}\right)=\frac{1}{1+e^{-\lambda {\text{net}}_{j}}},\\ \text{where}\;\;\;\;{\text{net}}_{j}=b_{j}+\underset{k}{\sum }O_{pk}W_{kj}, \end{array}$$

where *k* ranges over the input indices, *W*_*kj*_ is the weight on the connection from *k*-th input to *j*-th neuron, and *b*_*j*_ is the bias weight for the *j*-th output neuron.

To calculate the error signal at the output, the equations below can be used:

(2) $$\begin{array}{c} E=\frac{1}{2}\;\overset{N}{\underset{i=1}{\sum }}\;{\left(T_{pj}-O_{pj}\right)}^{2}, \end{array}$$

where *T*_*pj*_ is the target value of the *j*-th output neuron for pattern *p* and *O*_*pj*_ is the actual output value of the *j*-th output neuron for pattern *p*.

The backpropagation algorithm is based on the gradient descent optimization method [20–22]. By determining the derivative of error, we can update the network parameters. The output neuron error signal *d*_*pj*_ is determined as follows:

(3) $$\begin{array}{c} d_{pj}=\left(T_{pj}-O_{pj}\right)O_{pj}\left(1-O_{pj}\right). \end{array}$$

To calculate the error signal for each hidden neuron, the equations below can be used.

The hidden neuron error signal *δ*_*pj*_ is given by

(4) $$\begin{array}{c} \delta_{pj}=O_{pj}\left(1-O_{pj}\right)\underset{k}{\sum }\delta_{pk}W_{kj}, \end{array}$$

where *δ*_*pk*_ is the error signal of a postsynaptic neuron *k* and *W*_*kj*_ is the weight of the connection from *j*-th hidden neuron to the *k*-th postsynaptic neuron [21].

To calculate and apply weight adjustments, the equations below can be used:

(5) $$\begin{array}{c} W_{ji}\left(t+1\right)=W_{ji}\left(t\right)-\gamma \Delta W_{ji}\left(t\right)+\beta \left(W_{ji}\left(t\right)-W_{ji}\left(t-1\right)\right), \end{array}$$

where *γ* is the learning rate and *β* is the momentum. Here,

(6) $$\begin{array}{c} \Delta W_{ji}\left(t\right)=\delta_{pj}O_{pj}. \end{array}$$

### 2.2. Competitive Neural Network

**Figure 3:**
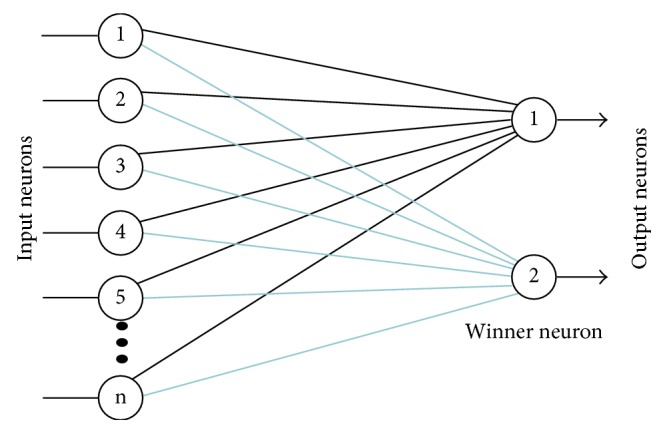
Competitive neural network.

The competitive neural network is a simple neural network that consists of two layers and uses an unsupervised learning algorithm for training. The inputs of the network are features, and the outputs are the classes. The input layer is fully connected to the output layer. Each connection between input and output layers is characterized by weight coefficients. In every epoch, the neurons in the output layer compete among themselves when input features are applied to the network input [23–25]. The competitive neural network (Figure 3) relies fundamentally on the Hebbian learning rule. The distinction is the following: in competitive learning, output neurons have to compete among themselves to get activated, and only one neuron is activated at any time, as compared to Hebbian learning where more than one neuron can be activated or fired at any time.

These networks use a “winner-takes-all” strategy, where only the weights connected to the winner neuron are updated in a particular epoch, while other weights are not updated [24, 25]. This learning process has the resultant effect of increasingly strengthening the correlation between the inputs and the corresponding winner neurons during learning.

When the patterns are supplied to the input layer, the neurons in the output layer compete among themselves to be activated [23–25]. The rules used to update the weights of these networks are given below. For output winner neuron *k*, we have

(7) $$\begin{array}{c} \Delta w_{kj}=\eta \left(x_{j}-w_{kj}\right), \end{array}$$

where *η* is the learning rate, *x*_*j*_ is the *j*-th input pattern, *w*_*kj*_ is the weight connection between *j*-th and *k*-th neurons, and Δ*w*_*kj*_ is the computed weight change.

If *k*-th output neuron loses at epoch *p*, then

(8) $$\begin{array}{c} \Delta w_{kj}=0. \end{array}$$

Weight update for *k*-th neuron at epoch (*p* + 1) is achieved using the following equation:

(9) $$\begin{array}{c} w_{kj}\left(p+1\right)=w_{kj}\left(p\right)+\Delta w_{kj}. \end{array}$$

### 2.3. Convolutional Neural Networks

**Figure 4:**
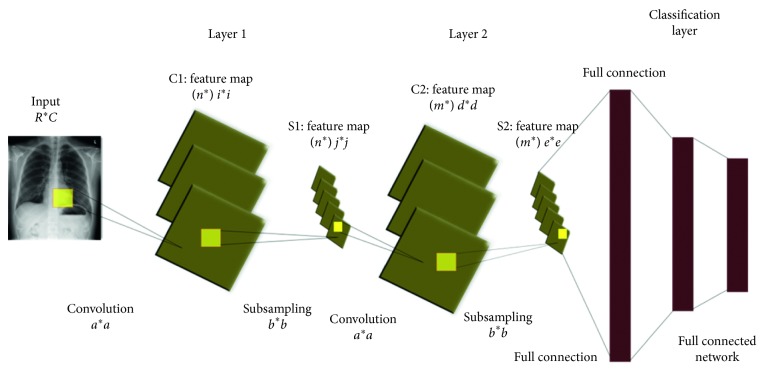
Convolutional neural network.

Deep learning is a machine learning method inspired by the deep structure of a mammal brain [26]. The deep structures are characterized by multiple hidden layers allowing the abstraction of the different levels of the features. In 2006, Hinton et al. developed a new algorithm to train the neuron layers of deep architecture, which they called greedy layerwise training [12]. This learning algorithm is seen as an unsupervised single layer greedily training where a deep network is trained layer by layer. Because this method became more effective, it has been started to be used for training many deep networks. One of the most powerful deep networks is the convolutional neural network that can include multiple hidden layers performing convolution and subsampling in order to extract low to high levels of features of the input data [27–30]. This network has shown a great efficiency in different areas, particularly, in computer vision [28], biological computation [29], fingerprint enhancement [30], and so on. Basically, this type of networks consists of three layers: convolution layers, subsampling or pooling layers, and full connection layers.  Figure 4 shows a typical architecture of a convolutional neural network (CNN). Each type of layer is explained briefly in the following sections.

#### 2.3.1. Convolution Layer

In this layer, an input image of size *R*∗*C* is convolved with a kernel (filter) of size *a*∗*a* as shown in Figure 4. Each block of the input matrix is independently convolved with the kernel and generated a pixel in the output. The result of the convolution of the input image and kernel is used to generate *n* output image features. Generally, a kernel of the convolution matrix is referred to as a filter while the output image features obtained by convolving kernel and the input images are referred to as feature maps of size *i*∗*i*.

CNN can include multiple convolutional layers, the inputs and outputs of next convolutional layers are the feature vector. There is a bunch of n filters in each convolution layer. These filters are convolved with the input, and the depth of the generated feature maps (*n*∗) is equivalent to the number of filters applied in the convolution operation. Note that each filter map is considered as a specific feature at a certain location of the input image [31–33].

The output of the *l*-th convolution layer, denoted as *C*_*j*_^(*l*)^, consists of feature maps. It is computed as

(10) $$\begin{array}{c} C_{i}^{\left(l\right)}=B_{i}^{\left(l\right)}+\overset{a_{i}^{\left(l-1\right)}}{\underset{j=1}{\sum }}K_{i,j}^{\left(l-1\right)}\;*\;C_{j}^{\left(l\right)}, \end{array}$$

where *B*_*i*_^(*l*)^ is the bias matrix and *K*_*i*,*j*_^(*l* − 1)^ is convolution filter or kernel of size *a*∗*a* that connects the *j*-th feature map in layer (*l* − 1) with the *i*-th feature map in the same layer. The output *C*_*i*_^(*l*)^ layer consists of feature maps. In (10), the first convolutional layer *C*_*i*_^(*l* − 1)^ is input space, that is, *C*_*i*_^(0)^=*X*_*i*_.

The kernel generates feature map. After the convolution layer, the activation function can be applied for nonlinear transformation of the outputs of the convolutional layer:

(11) $$\begin{array}{c} Y_{i}^{\left(l\right)}=Y\left(C_{i}^{\left(l\right)}\right), \end{array}$$

where *Y*_*i*_^(*l*)^ is the output of the activation function and *C*_*i*_^(*l*)^ is the input that it receives.

Typically used activation functions are sigmoid, tanh, and rectified linear units (ReLUs). In this paper, ReLUs which is denoted as *Y*_*i*_^(*l*)^=max(0, *Y*_*i*_^(*l*)^) are used. This function is popularly used in deep learning models due to its help in reducing the interaction and nonlinear effects. ReLU converts the output to 0 if it receives a negative input, while it returns the same input value if it is positive. The advantage of this activation function over other functions is the faster training because of the error derivative, which becomes very small in the saturating region; therefore, the updates of the weights almost vanish. This is called the vanishing gradient problem.

#### 2.3.2. Subsampling Layer

The main aim of this layer is to spatially reduce the dimensionality of the features maps extracted from the previous convolution layer. To do so, a mask of size *b*∗*b* is selected as shown in Figure 4, and the subsampling operation between the mask and the feature maps is performed. Many subsampling methods were proposed such as averaging pooling, sum pooling, and maximum pooling. The most commonly used pooling is the max pooling, where the maximum value of each block is the corresponding pixel value of the output image. Note that a subsampling layer helps the convolution layer to tolerate rotation and translation among the input images.

#### 2.3.3. Full Connection

The final layer of a CNN is a traditional feedforward network with one or more hidden layers. The output layer uses Softmax activation function:

(12) $$\begin{array}{c} y_{i}^{\left(l\right)}=f\left(z_{i}^{\left(l\right)}\right),\;\;\mathrm{where}\;\;z_{i}^{\left(l\right)}=\overset{m_{i}^{\left(l-1\right)}}{\underset{i=1}{\sum }}w_{i,j}^{\left(l\right)}y_{i}^{\left(l-1\right)}, \end{array}$$

where *w*_*i*,*j*_^(*l*)^ are the weights that should be tuned by the complete fully connected layer in order to form the representation of each class and *f* is the transfer function which represents the nonlinearity. Note that the nonlinearity in the fully connected layer is built within its neurons, not in separate layers as in convolutions and pooling layers.

After finding output signals, the training of the CNN is started. Training is performed using the stochastic gradient descent algorithm [34]. The algorithm estimates the gradients using a single randomly picked example from the training set. As a result of training, the parameters of CNN are determined.

## 3. Simulations

In this section, the simulations of the above networks are described. Note that the BPNN and CpNN networks are trained using 620 out of 1000 images, and the rest is used for testing. The CNN is trained using 70% of 120,120 available data, and 30% are used for testing. The input images are of size 32 × 32 for the sake of reducing computation cost.

### 3.1. Simulation of Chest Diseases Using BPNN

Backpropagation neural network is based on a supervised learning algorithm, and they are very important and useful in pattern recognition problems [17, 19, 35]. The training of backpropagation networks includes the update of parameters in order to produce good classification results. Hence, in this paper, several experiments were conducted such that significantly accurate results can be obtained. For this aim, different number of hidden neurons, learning rate, and momentum are applied for obtaining better classification result.

**Figure 5:**
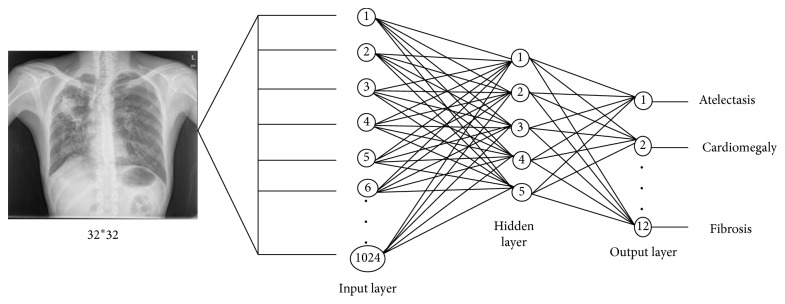
Backpropagation neural network.

The architecture of the designed backpropagation neural network for the image of size 32 × 32 is described in Figure 5.

Since the backpropagation network uses a supervised learning algorithm, it is, therefore, necessary that the training data could be labelled. The used training data have been labelled according to the 12 classes presented in the classification task. In training stage, different number of hidden neurons, learning rate, and momentum were experimented for obtaining better classification result.  Table 1 presents the used architectures of BPNN, denoted as BPNN1, BPNN2, BPPN3, and BPNN4.

**Figure 6:**
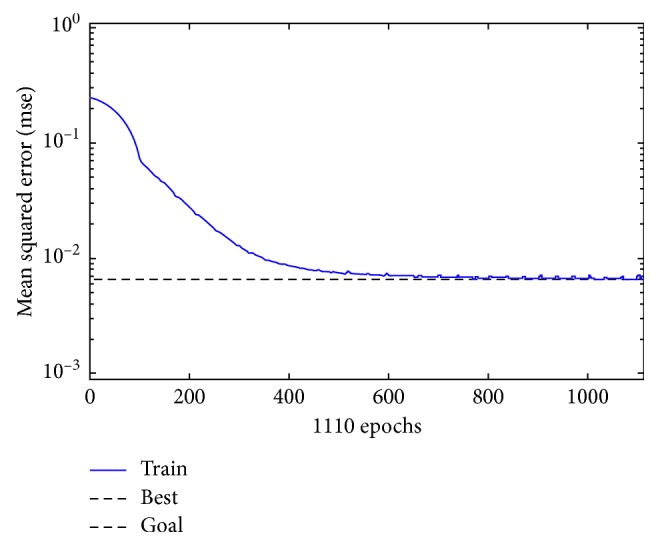
Learning curve for BPNN2.

Since there are 12 classes, 12 neurons have been used in the output layer of the network. The learning curve of BPNN2, which is the network with lowest achieved MSE (Table 1), is shown in Figure 6.

### 3.2. Simulation of Chest Diseases Using Competitive Neural Network (CpNN)

**Figure 7:**
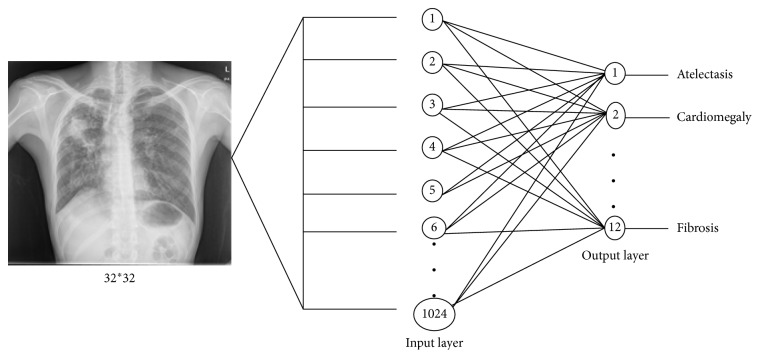
Competitive neural network.

In this section, a competitive neural network using an unsupervised learning algorithm is used for classification of chest diseases. Leveraging on the fact that such networks do not need manual labelling of training data, they save time for the labelling process.  Figure 7 shows the architecture of the network used in this paper.

The competitive neural network has two layers designated for the input and output signals. The images are fed as input to the network, and the output neurons learn unique attributes or patterns of the images that differentiates one class from the others. The number of input neurons is 1024 (input image pixels), and the number of output neurons is 12 (number of output classes).

The training parameters of the networks used in this paper are given in Table 2. These competitive networks are trained using 32 × 32 pixels images. Since the network uses an unsupervised learning algorithm, there is no mean squared error goal to minimize.

### 3.3. Simulation of Chest Diseases Using Convolutional Neural Networks

In this section, the design of the convolutional neural network employed for the chest X-ray medical images are presented. The suitable values of learning parameters of the network are determined through experiments. Note that out of the obtained 120,120 images, 70% are used for training and 30% are used for validating the network.

The input images of the network are of size 32 × 32. The outputs are 12 classes. The proposed CNN includes 3 hidden layers.  Table 3 shows the structure of the CNN and its learning parameters. Here, “Conv” represents a convolution layer, “BN” represents batch normalization, “FM” represents feature maps, and “FC” represents fully connected layer. Note that the filters of size 3 × 3 are used in all convolution operations with padding, while all pooling operations are performed using max pooling windows of size 2 × 2.

During simulation, the size of available training data and system specifications for constructing a model were taken into consideration. Thus, dropout training schemes and a batch normalization were employed, and the improvement in model generalization was achieved [24, 25]. Note that a minibatch optimization of size 100 via stochastic gradient descent is employed [34] for training. In addition, a learning rate of 0.001 and 40,000 iterations are used for training of the CNN model.

**Figure 8:**
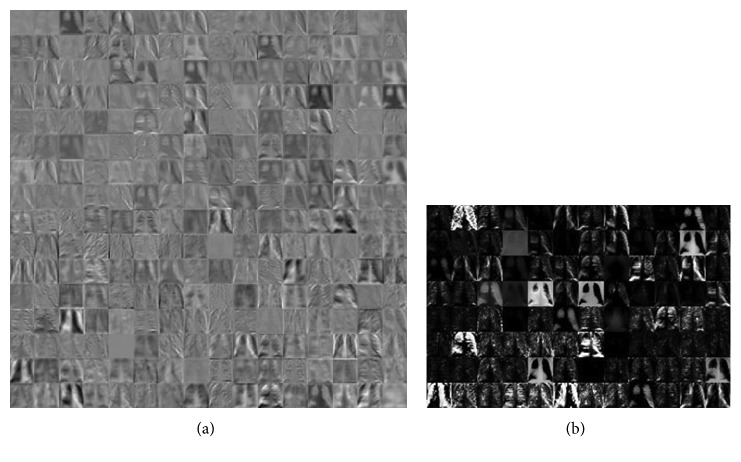
Learned filters: (a) convolution layer 1 and (b) pooling layer 1.

The extraction of different levels of features of chest X-ray images in both convolution and pooling layer 1 is given in Figure 8.  Figure 8(a) shows the learned filters (or kernels) at convolution layer 1 and Figure 8(b) at the pooling layer of the CNN.

## 4. Discussion of Results

The overall performances of the BPNN and CpNN are tested using 380 images.  Table 4 shows the recognition rates obtained for the backpropagation networks using 32 × 32 pixels as the input image size.

It can be seen from the table that all the trained backpropagation neural networks (BPNNs) have different training and testing performances. BPNN2 achieved the highest recognition rate for both training and testing datasets compared to the other networks, that is, 99.19% and 89.57%, respectively.

Competitive neural networks that use an unsupervised learning algorithm were also trained and tested using the same images. These networks are faster to train, considering that they have no desired outputs and therefore no error computations and back-pass of error gradients for weights update. The simulation results of the competitive networks using different learning rate and the number of maximum epochs are given in Table 5.

From the table, it can be seen that CpNN2 has the highest recognition rates for both training and test data. Furthermore, it can be seen that CpNN3 has a higher recognition rate than CpNN2 for the training data. Its performance on the test data is lower than CpNN2; that is, it can be stated that CpNN3 has lower generalization power as compared to CpNN2.

Furthermore, the convolutional neural network (CNN) designed for this classification task is also tested using 30% of the available chest X-ray images, and the results are shown in Table 6.

Overall, the performance of the three employed networks in terms of recognition rate, training time, and reached mean square error (MSE) is described in Table 7.

**Figure 9:**
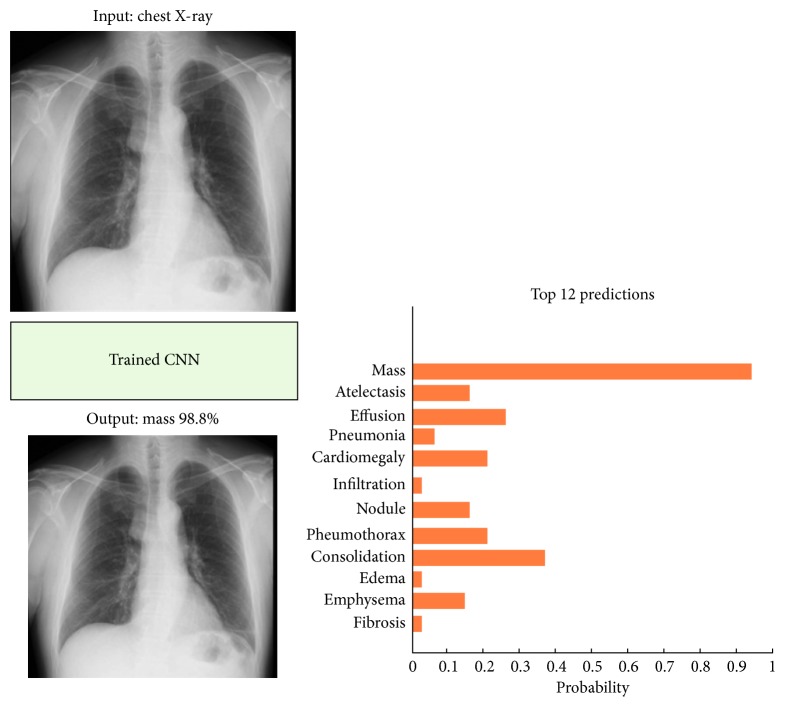
CNN final classification of chest X-rays with classes probabilities.

As shown in Tables 4 and 5, the networks behave differently during training and testing, and this is obviously due to the difference in the structures, working principles, and training algorithms of the three employed networks. Also in Table 7, the CNN has achieved the highest recognition rate for training and testing data, compared to other employed networks. In contrast, this outperformance of CNN over other networks requires longer time and a larger number of learning iterations than that of BPNN2 and CpNN2. Moreover, it can be seen that the three networks have achieved a low MSE, whereas the CNN scored the lowest (0.0013). Furthermore, it is noted that the time needed for the CNN to converge is roughly higher than that of BPNN2 and CpNN2. Consequently, this is due to the depth of the structure of a convolutional neural network, which normally requires a long time, in particular, when the number of inputs is large. Nonetheless, this deep structure is the main factor in achieving a higher recognition rate compared to other networks such as BPNN and CpNN. Lastly, Figure 9 shows an example of the CNN testing paradigm. The networks first take a chest X-ray as an input and output the probabilities of the classes.

A comparison of the developed networks with some earlier works is shown in Table 8. Firstly, it is seen that shallow (traditional) networks (BPNN and CpNN) could not achieve high recognition rates compared to other deep networks, which is obviously due to their deficiency in extracting the important features from input images. Moreover, it is noticed that the proposed deep convolutional neural network (CNN) achieved a higher recognition rate than other earlier research work such as CNN with GIST features [36]. The transfer learning-based networks are also used for chest X-rays classification such as VGG16 [37] and VGG19 [37]. They have gained lower generalization capabilities compared to the proposed network. These pretrained models [37] have very powerful features extraction capabilities since they were trained using a huge database, Image Net [38]. Note that, we compared the researches that provided explicitly achieved accuracies. The obtained results can show that applying deep CNNs to the problem of chest X-ray diseases is promising in a way that similar or confusing diseases could be correctly classified with good recognition rates.

## 5. Conclusion

In this paper, convolutional neural network (CNN) is designed for diagnosis of chest diseases. For comparative analysis, backpropagation neural network (BPNN) and competitive neural network (CpNN) are carried out for the classification of the chest X-ray diseases. The designed CNN, BPNN, and CpNN were trained and tested using the chest X-ray images containing different diseases. Several experiments were carried out through training of these networks using different learning parameters and a number of iterations. In both backpropagation and competitive networks, it was observed that the input image of size 32 × 32 pixels showed good performance and achieved high recognition rates. Based on recognition rates, the backpropagation networks outperformed the competitive networks. Moreover, the competitive networks did not require manual labelling of training data as it was carried out for the backpropagation network. Furthermore, a CNN was also trained and tested using a larger dataset which was also used for training and testing of BPNN and CpNN. After convergence, it was noticed that the CNN was capable of gaining a better generalization power than that achieved by BPNN and CpNN, although required computation time and the number of iterations were roughly higher. This outperformance is mainly due to the deep structure of CNN that uses the power of extracting different level features, which resulted in a better generalization capability. The simulation result of proposed CNN is also compared with other deep CNN models such as GIST, VGG16, and VGG19. These networks have lower generalization capabilities and accuracies compared to the proposed network. The obtained results have demonstrated the high recognition rates of the proposed CNN.

## Figures and Tables

**Table 1:** Training parameters for backpropagation networks (32 × 32 input pixels).

Networks	BPPN1	BPNN2	BPNN3	BPNN4
Training samples	620	**620**	620	620
Hidden neurons	20	**35**	45	60
Learning rate	0.010	**0.0045**	0.300	0.15
Momentum rate	0.040	**0.0072**	0.0504	0.0619
Activation function	Sigmoid	**Sigmoid**	Sigmoid	Sigmoid
Epochs	1000	**1000**	1256	1374
Training time (sec)	148	**156**	184	193
Mean squared error	0.0077	**0.0025**	0.0056	0.0096

**Table 2:** Training parameters for competitive neural network (32 × 32 input pixels).

Networks	CpNN1	CpNN2	CpNN3
Training samples	**620**	620	620
Learning rate	**0.0036**	0.05	0.1
Maximum epochs	**1000**	2000	4000
Training time (sec)	**300** **secs**	434 secs	468

**Table 3:** CNN training parameters.

Layers	Description	Values
Input layer	Input image	32 × 32 × 1 images with “zerocenter” normalization

Hidden layer 1	Conv1 + BN + ReLu	16 feature maps of size 10 × 10
	Pool1	2 × 2 kernel size with stride of 2

Hidden layer 2	Conv2 + BN + ReLu	32 feature maps of size 10 × 10
	Pool2	2 × 2 kernel size with stride of 2

Hidden layer 3	Conv3 + BN + ReLu	64 feature maps of size 10 × 10
Classification layer	FC	2 fully connected layers
	Softmax	2 units

**Table 4:** Recognition rates for BPNNs on training and validation data (32 × 32 pixels).

Network models	Training data (70%)	Validation data (30%)
BPNN1	92.74%	87.42%
**BPNN2**	**99.19%**	**89.57%**
BPNN3	97.32%	84.36%
BPNN4	98.10%	85.24%

**Table 5:** Recognition rates for CpNNs using training and validation data (32 × 32 pixels).

Network models	Training data (70%)	Validation data (30%)
CpNN1	84.21%	81.40%
**CpNN2**	**85.23%**	**84.71%**
CpNN3	86.57%	76.25%

**Table 6:** Recognition rates for CNNs on training and validation data (32 × 32 pixels).

Network model	Training data (70%)	Validation data (30%)
CNN	100%	92.4%

**Table 7:** Performance of the BPNN, CpNN, and CNN.

Network models	Training time	Recognition rate	Reached MSE	Maximum number of iterations
BPNN2	630 secs	80.04%	0.0025	5000
CpNN2	300 secs	89.57%	0.0036	1000
CNN	2500 secs	92.4%	0.0013	40,000

**Table 8:** Results comparison with earlier works.

Parameters	CNN	BPNN2	CpNN2	CNN with GIST [36]	VGG16 [37]	VGG19 [37]
Number of images	120,120	1000	1000	637	8100	8100
Accuracy	92.4%	80.04%	89.57%	92%	86%	92%

## Data Availability

The data used to support the findings of this study are available from the corresponding author upon request.
